# A Flattest Constrained Envelope Approach for Empirical Mode Decomposition

**DOI:** 10.1371/journal.pone.0061739

**Published:** 2013-04-23

**Authors:** Weifang Zhu, Heming Zhao, Dehui Xiang, Xinjian Chen

**Affiliations:** School of Electronics and Information Engineering, Soochow University, Suzhou, China; Cuban Neuroscience Center, Cuba

## Abstract

Empirical mode decomposition (EMD) is an adaptive method for nonlinear, non-stationary signal analysis. However, the upper and lower envelopes fitted by cubic spline interpolation (CSI) may often occur overshoots. In this paper, a new envelope fitting method based on the flattest constrained interpolation is proposed. The proposed method effectively integrates the difference between extremes into the cost function, and applies a chaos particle swarm optimization method to optimize the derivatives of the interpolation nodes. The proposed method was tested on three different types of data: ascertain signal, random signals and real electrocardiogram signals. The experimental results show that: (1) The proposed flattest envelope effectively solves the overshoots caused by CSI method and the artificial bends caused by piecewise parabola interpolation (PPI) method. (2) The index of orthogonality of the intrinsic mode functions (IMFs) based on the proposed method is 0.04054, 0.02222±0.01468 and 0.04013±0.03953 for the ascertain signal, random signals and electrocardiogram signals, respectively, which is lower than the CSI method and the PPI method, and means the IMFs are more orthogonal. (3) The index of energy conversation of the IMFs based on the proposed method is 0.96193, 0.93501±0.03290 and 0.93041±0.00429 for the ascertain signal, random signals and electrocardiogram signals, respectively, which is closer to 1 than the other two methods and indicates the total energy deviation amongst the components is smaller. (4) The comparisons of the Hilbert spectrums show that the proposed method overcomes the mode mixing problems very well, and make the instantaneous frequency more physically meaningful.

## Introduction

EMD is a new adaptive data analysis method for analyzing nonlinear and non-stationary data, which was proposed by Huang et al. in 1998 [1]. It decomposes the signal into several basic components called intrinsic mode functions (IMFs), and a residual understood as the signal trend. IMFs are supposed to be zero mean functions modulated in amplitude and/or frequency, i.e., IMFs are truly AM/FM monocomponent signals. Any complex signal can be decomposed by using the EMD method into a series of IMFs. The decomposing is generated at each scale from fine to coarse through an iterative procedure called sifting process. Combined with the Hilbert transform, it can provide a powerful tool in time-frequency signal analysis and processing.

Although the EMD is still in developing, it has been successfully applied in various science and engineering fields [2]–[7]. However, besides the lack of a firm mathematical foundation, the EMD method also faces a number of problems in the implementations and applications of the algorithm, such as envelope estimation, stopping criterion, mode mixing, end issue, etc. Among these problems, the envelope estimation is the most important one and needs to be paid specific attention. The upper and lower envelopes play a crucial role in the EMD algorithm. The classic envelope estimation is implemented by CSI. As shown in some papers [1], [8], [9], the CSI algorithm produced acceptable results in many situations. However, an overshoot problem often occurs for the CSI algorithm because the CSI curve is the second order derivable and usually too “smooth”. Overshoot not only shifts the mean value of the upper and lower envelopes, but also has an adverse impact on the decomposition of IMFs. It needs further study and improvement.

Many approaches have been proposed to substitute the CSI, such as the B-spline interpolation [10], the segment power function interpolation [11], the GA-based optimization of the piecewise polynomials interpolation [12] and the piecewise parabola interpolation (PPI) [13]. Chen et al. [10] proposed a B-spline algorithm for EMD. They believed that the IMFs obtained could be represented by B-splines according to this algorithm. The instantaneous frequency of the IMFs is determined by the Hilbert transform of B-splines. Therefore, it is desirable to study basic properties of the Hilbert transform of B-splines. They proposed a recursive algorithm for the computation of the Hilbert transform of B-splines which they believed may offer convenience in computation. However, the B-spline interpolation approach is only introduced to improve the analytical performance. Qin et al. [11] proposed a segment power function method based on the principle of parabola parameter spline interpolation method which has intuitive geometric meaning. The segment power function approach has a conclusion that it can fit smooth and flexible envelopes when the power index 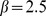 through experiments. But how to determine a good value for 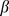 is needed to be further investigated. Kopsinis et al. [12] proposed a GA-based optimization of EMD in both the interpolation points and the piecewise interpolating polynomials. Their research showed that there are specific extrema, which are related to the signal that is to be extracted and are able to lead to much improved decomposition performance if the extrema of EMD are set to them. As a result, EMD can be understood as a procedure that attempts to iteratively converge to those optimized extrema. A type of Hermitian interpolants had an “envelope like” behavior. However, the GA-based optimization of both the interpolation points and the piecewise polynomials approach is too complicated. Xu et al. [13] proposed a PPI approach for EMD. The envelope based on PPI method has a simple analytical expression, so that the behavior of the sifting process can be seen more clearly. The piecewise parabola interpolation approach may overcome the overshoots well, but it may cause artificial bends at junctions of the adjacent curves. According to the methods mentioned above, there are still some shortcomings in the existing envelope fitting methods and they need to be further studied and improved.

In this paper, a new envelope fitting approach for EMD, called the flattest constrained piecewise cubic Hermite interpolation (FC-PCHI), is proposed which is based on the optimization of the piecewise cubic Hermite interpolation (PCHI). The proposed method consists of PCHI and the optimization of the derivatives of the interpolate points. The proposed method was tested on three different types of data: ascertain signal, random signals and real electrocardiogram (ECG) signals. The experimental results proved that the new method can solve the overshoots caused by CPI and the artificial bends caused by PPI effectively, and let the IMFs more accurate and more reasonable. This method can overcome mode mixing well, which is one of the major drawbacks of the original EMD.

The rest of this paper is organized as follows. Section 2 describes the background of EMD and the FC-PCHI method, which is based on analysis and comparison of the interpolation methods. Section 3 shows the experimental results from using different interpolation methods. Section 4 provides a discussion and conclusion.

## Methods

### 2.1 Background of EMD

**Figure 1:**
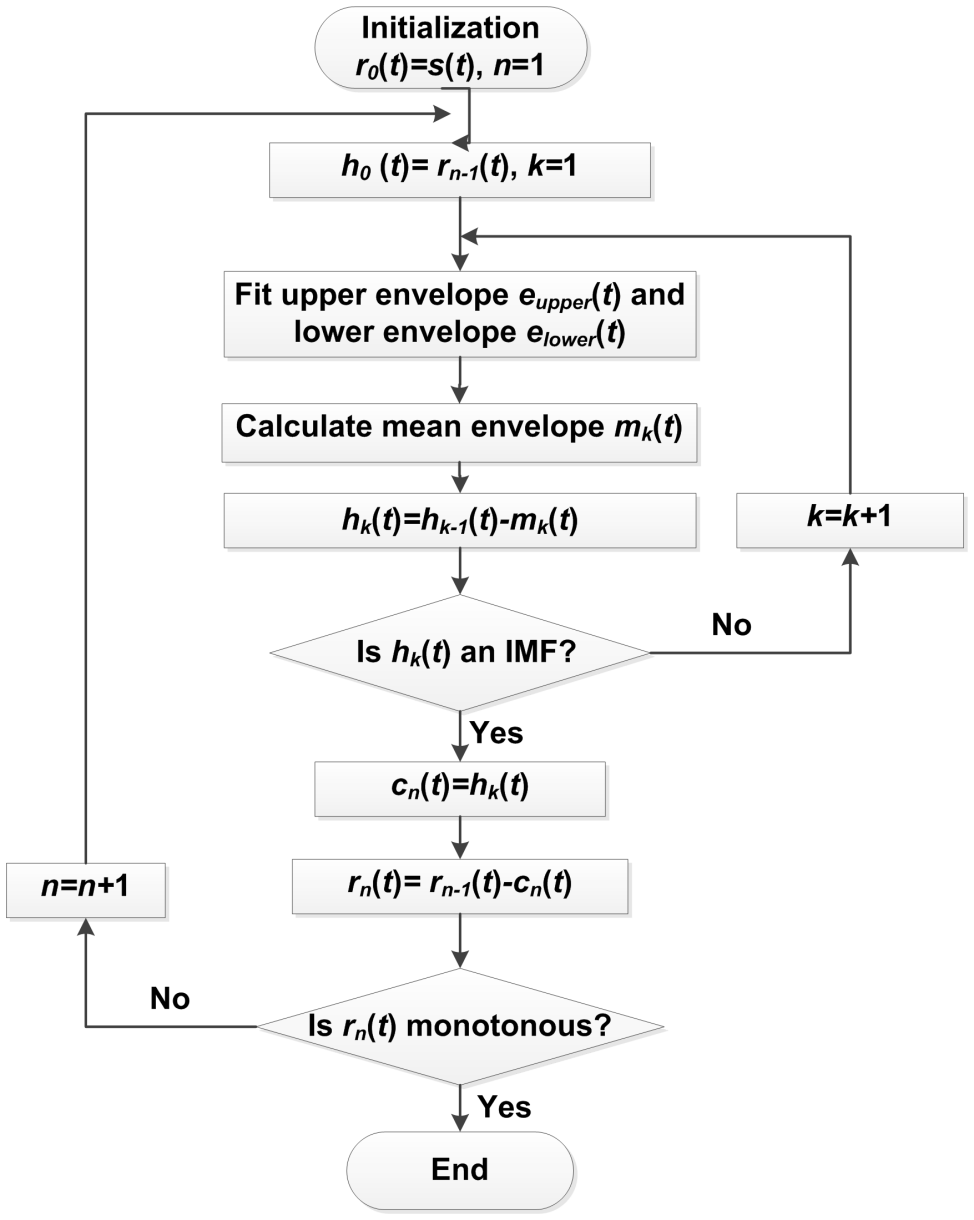
The flow chart of EMD.

The main idea of the EMD is to decompose signals into oscillatory components from the fastest (high frequencies) to the lowest ones (low frequencies). Following Huang et al. [1], for a given signal denoted as 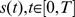, the flow chart of EMD algorithm is shown in Figure 1.

At the end of the decomposition, the signal 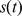 is represented as:
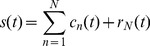
(1)where 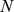 is the number of IMF components and 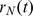 is the final residual. If we refer to 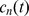 as the *n*th-order IMF, low-order IMFs capture fast oscillation modes while high-order IMFs typically represent slow oscillation modes.

### 2.2 Introduction of the Interpolation Methods

The common linear interpolation method only considers the adjacent two points and is simply constructed. However, the fitted curve is not smooth and may cause cusps at the interpolation points. The interpolation accuracy of the low order polynomials interpolation method is relatively poor. But when the order of the interpolation polynomials increases, the Lunge phenomenon [14] will occur. If the number of the interpolation points is too large, the piecewise low order polynomials interpolation method should be used [14].

The CSI method is a widely used interpolation method, which uses piecewise polynomials as interpolation functions. To keep the smoothness of the fitted curve, the first and second order derivatives of the interpolation points must exist. However, overshoots often occur when using CSI method because of its second-order smoothness. The overshoots shift the mean value curve of the upper and lower envelopes. Another hand, the CSI method is a global interpolation method, which means if one of the interpolation points changes, the whole fitted curve will change. These issues caused the CSI method with global property may not be suitable to analyze the non-stationary signals emphasized on the local characteristics.

**Figure 2:**
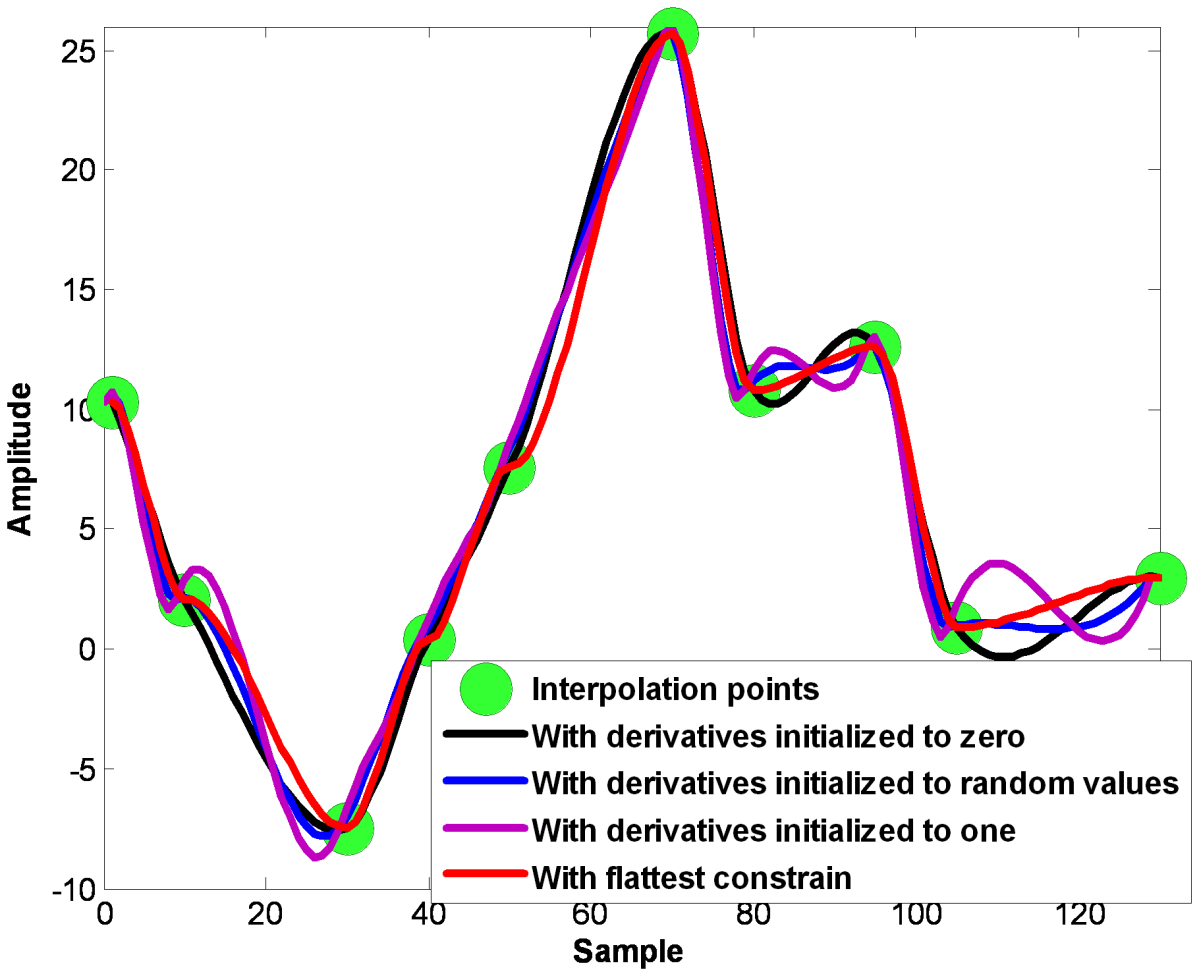
PCHI curves with different derivatives.

The PCHI is an effective interpolation method which can express local information of signals effectively [14]. Let the original curve on the interval [a, b] be denoted by 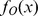, and the corresponding PCHI function be denoted by 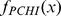. Let the interpolation points be remarked as 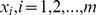, and 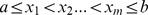. PCHI method requires 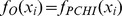 and 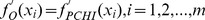, respectively. So PCHI functions are cluster functions with the first order derivatives of the interpolation points, and their performance depend on these derivatives. Figure 2 shows the PCHI functions with different derivatives. Akima interpolation [15] is a special case of the PCHI method, whose derivatives depend on 5 adjacent interpolation points. It can weaken the overshoot well, but not suitable for processing the short signal.

To overcome the overshoots caused by the CSI functions, the interpolation functions should be flattened i.e., the difference between the minimum and maximum should be smaller. When the difference reaches the smallest one, the corresponding curve should be regarded as the flattest one. Therefore, it is reasonable to require that the PCHI functions should be not only smooth, but also the flattest one with the smallest ups and downs among the numerous smooth curves. So how to optimize these first order derivatives in the PCHI method, and make the PCHI curve be the flattest one is the target of the proposed method.

### 2.3 The Proposed FC-PCHI Method

The PCHI functions are cluster functions with the first order derivatives of the interpolation points. The key of optimization on PCHI method is the derivatives. Taking the upper envelope as an example, we describe our approach in details as follows.

Let 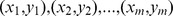 be maximum points in one sifting process of the EMD. Let 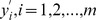 be the first order derivatives of these points. On each interval 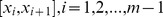, the PCHI curve can be written as
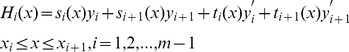
(2)where



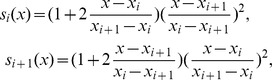


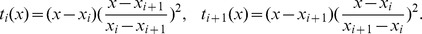



The fitted upper envelope can be written as 
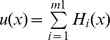
. On each interval 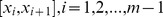, compute the maximum 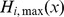 and the minimum 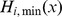. Then, the difference between extrema on the total interval 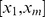 can be written as
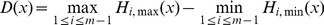
(3)


Obviously, 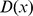 is also the function of parameters 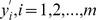 and can be written as 
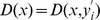
.

The problem to find the flattest PCHI curve equals to the problem to find the optimized 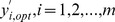 which satisfies the following expression.
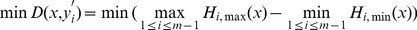
(4)


**Figure 3:**
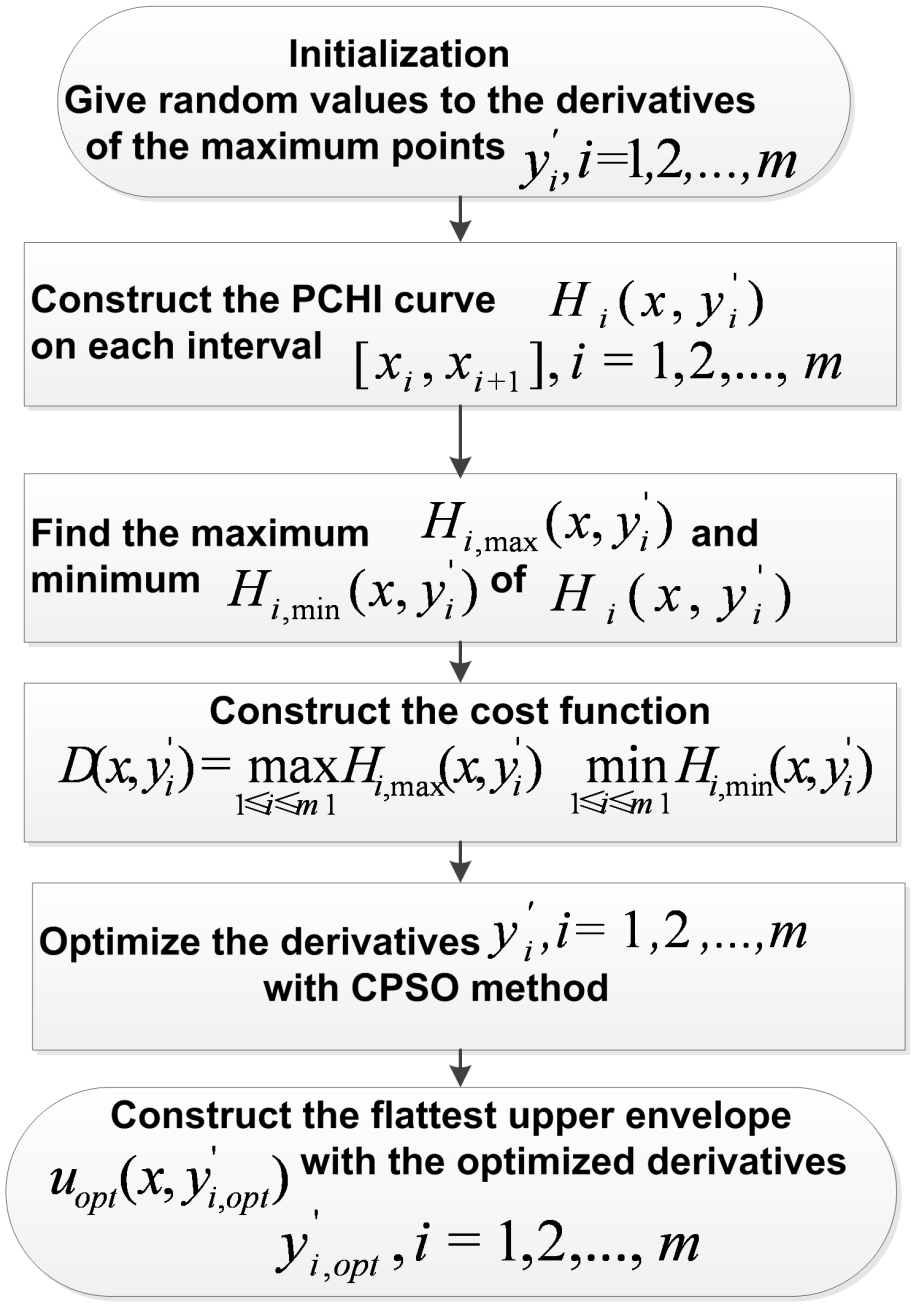
The flow chart of the proposed FC-PCHI method.

Equation (4) is an unconstrained nonlinear planning on parameters 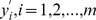. Because there are extrema computations in the cost function (3), the regular Lagrange optimization is not suitable. There are many optimization methods such as chaos particle swarm optimization (CPSO) method, simplex substitution, pattern search method, conjugate direction method etc. can be used. CPSO algorithm is derived from the simulation of birds’ foraging behavior [16] and is an optimization tool based on iterations. The property of ergodicity of chaos motion is used to avoid the locally optimal solution and try to find the global optimal solution. We adopt the CPSO method to get the optimized derivatives 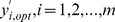 due to its simple concept, easy implementation, quick convergence, unique ergodicity and special ability to avoid being trapped in local optima. Then with the optimized derivatives 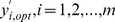, the flattest upper envelope 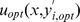 can be fitted with equation (2). The flow chart is shown in Figure 3.

Similarly, we can find the flattest lower envelope 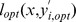. Then the flattest mean envelope 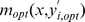 can be calculated as the average of 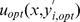 and 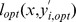. Let the flattest mean envelopes 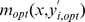 replaces the original mean envelope estimated with CSI method in the sifting process (SP), and other steps of the EMD keep unchanged. Then, we can get a series of IMFs based on the proposed FC-PCHI method.

## Results

The proposed method was tested on three types of signals: ascertain and stationary signal, 100 random Gaussian signals and 200 segments of real ECG signals.

### 3.1 Ascertain Signal

To evaluate the performance of the proposed FC-PCHI envelope, the proposed method is compared with the CSI envelope and the PPI envelope. Let us first consider the following signal,
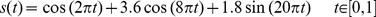
(5)


**Figure 4:**
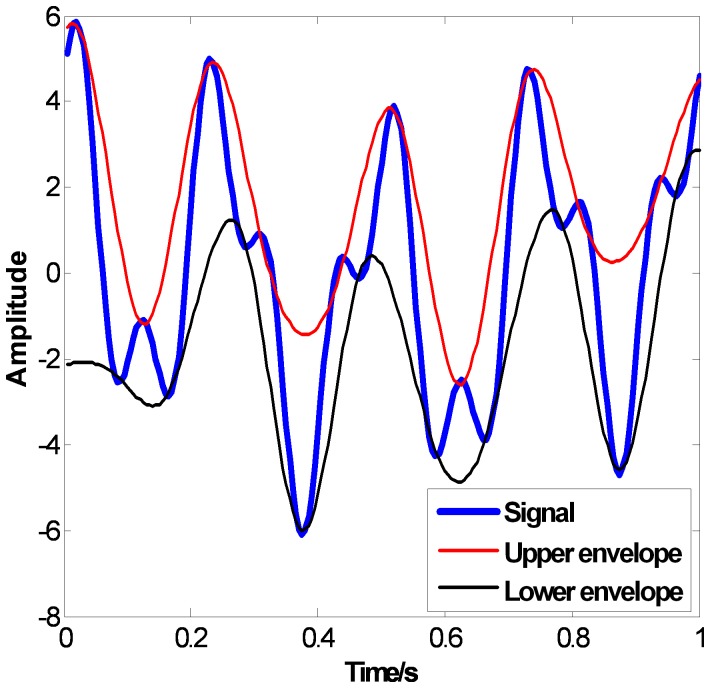
the CSI envelopes.

**Figure 5:**
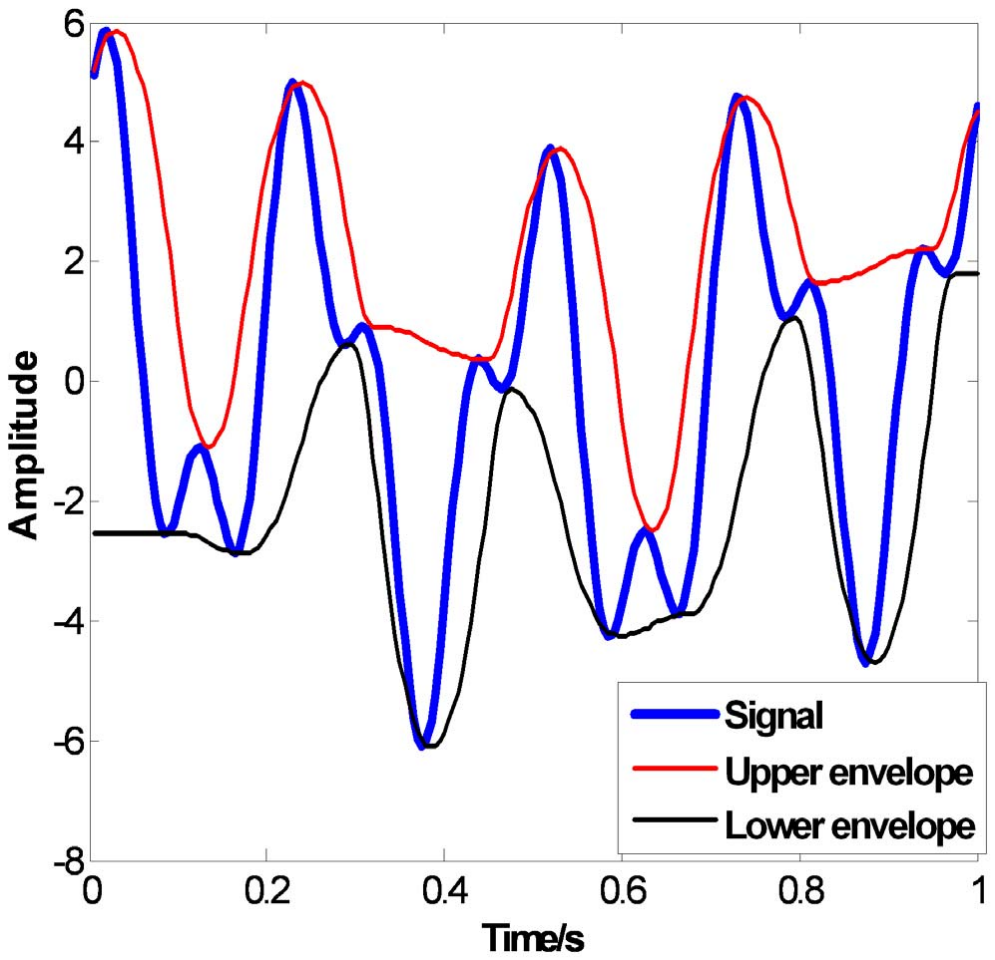
the PPI envelopes.

**Figure 6:**
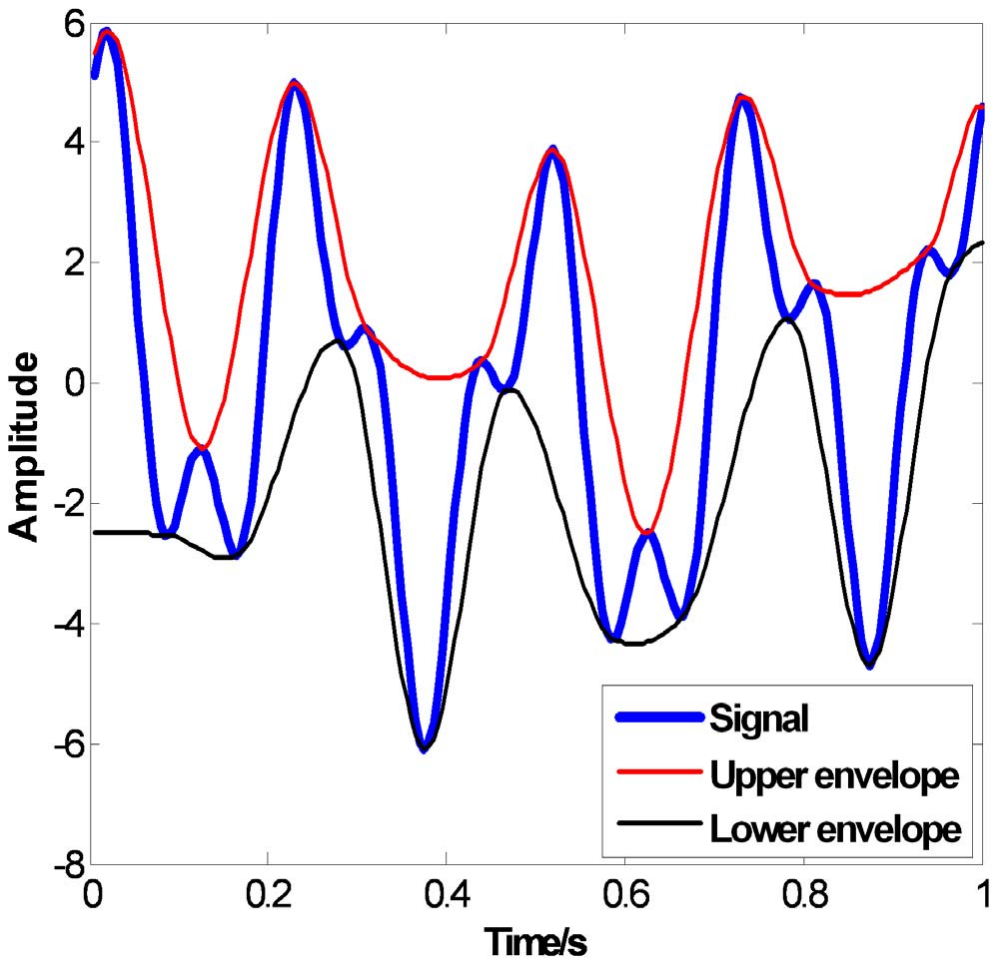
the FC-PCHI envelopes.

The CSI envelopes are shown in Figure 4. As we can see from Figure 4, overshoots occur obviously when 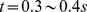 and 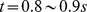 in the upper envelope, and when 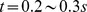 and 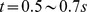 in the lower envelope. The PPI envelopes are shown in Figure 5, which overcome the overshoots well. But artificial bends occur when 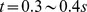 and 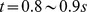 in the upper envelope and at the both ends of the lower envelope, which mean the PPI envelopes are flexible but not smooth enough. Compared to CSI and PPI, the proposed FC-PCHI envelopes are very smooth and no overshoots occur, which are shown in Figure 6.

**Figure 7:**
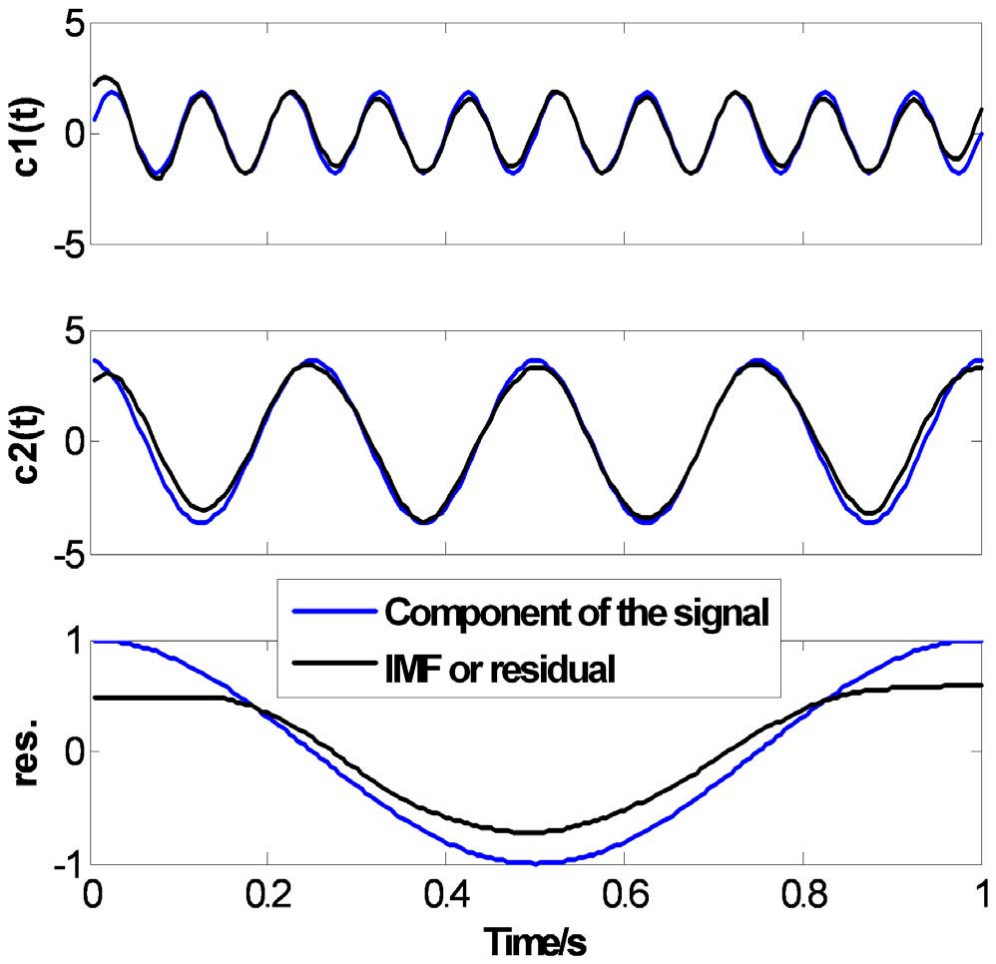
Ascertain signal’s CSI EMD results.

**Figure 8:**
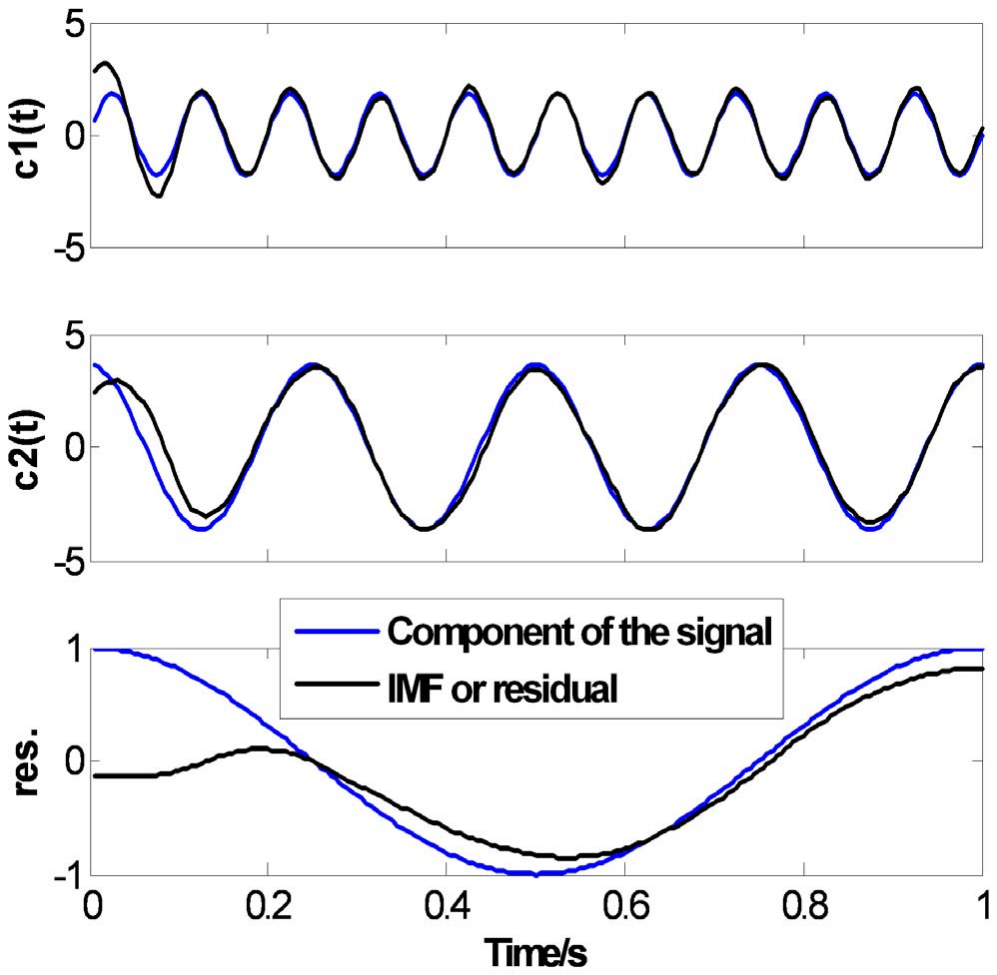
Ascertain signal’s PPI EMD results.

**Figure 9:**
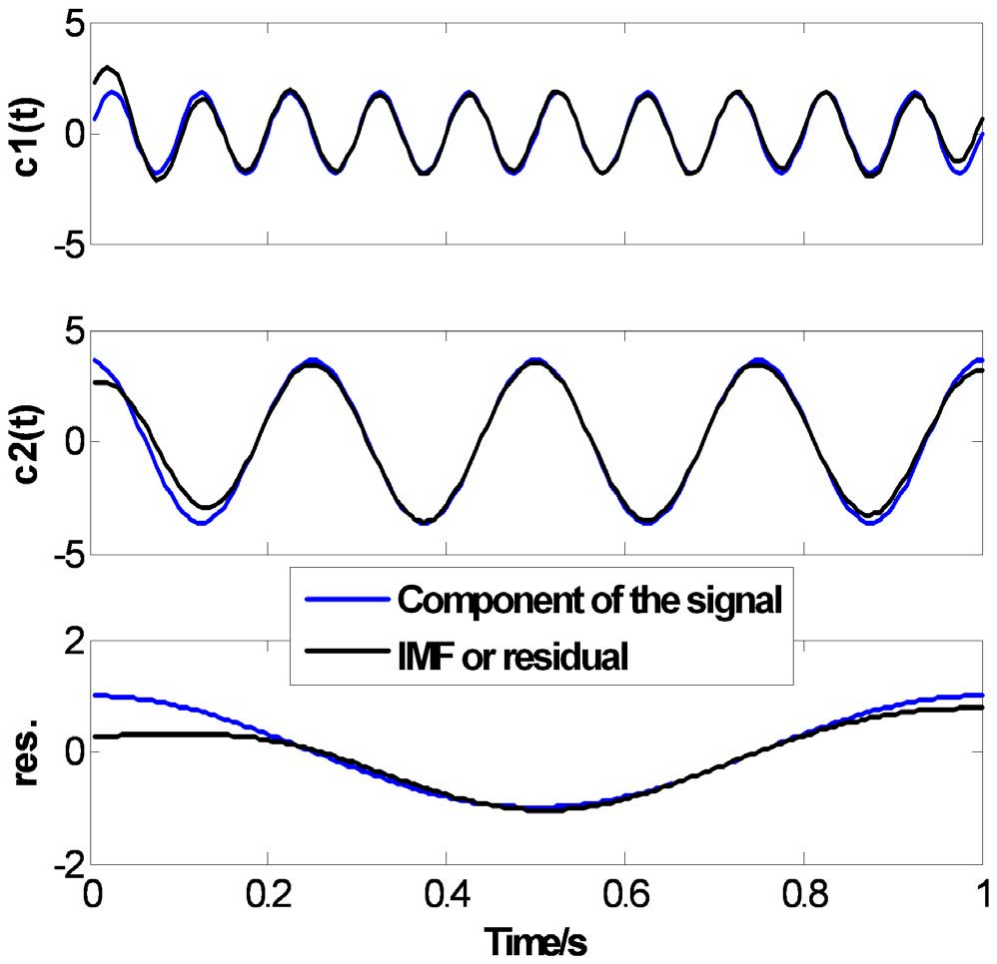
Ascertain signal’s FC-PCHI EMD results.

Figures 7, 8 and 9 show the EMD results of 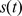
using CSI method, PPI method and the proposed FC-PCHI method, respectively. In the figures, the dotted lines represent the components of the original signal, and the solid lines represent the IMFs or residual through EMD. For the comparability of the results, the extreme points extend 2 points at the both ends according to the mirror extending regulation [17], which can weaken the end issue well, and the stopping criterion in literature [1] is used and 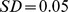. As can be seen in Figures 7, 8 and 9, these three methods can all decompose the complex signal into 3 mono-component signals. There are end issues in the first IMF 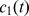 which cause mild swings at the both ends. Because the signal 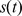 is short, the swings spread inward quickly during the sifting process. There are obvious distortions at both ends of 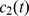 and especially at both ends of the residual. But as shown in Figures 7 and 8, there are obvious distortions inside 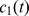 and 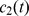 with the CSI method and the PPI method. As shown in Figure 9, 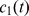 and 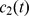 with the proposed FC-PCHI method fit the components of the signal 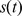 better except the ends.

To measure the index of orthogonality (IO) [1] and index of energy conservation (IEC) [10], the following equation (6) and equation (7) are used, respectively.



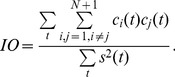
(6)




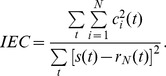
(7)where the residual 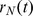 is treated as the (*N*+1)-th IMF. If the IO is close to zero, then the IMFs and the residual are close to orthogonal. The IEC reflects the fact that the sum of IMFs represents the signal minus the trend given in the residual. If the IEC is close to one, then the decomposition is close to lossless.

Based on the test results summarized in Table 1, we can see that the proposed FC-PCHI method indeed have much better performances than the CSI method and the PPI method, the performance of IO and IEC is 0.04054 and 0.96293, respectively. This indicates that the IMFs based on the proposed FC-PCHI method keep much better orthogonal and the total energy deviation amongst the components is the smallest. However, the IEC did not equal to one, which may be caused by end issues or stopping criterions.

**Table 1:** The comparison of IO and IEC results for ascertain signal.

	CSI method	PPI method	FC-PCHI method
IO	0.09951	0.06956	0.04054
IEC	0.85849	0.87437	0.96193

### 3.2 Random Signal

**Figure 10:**
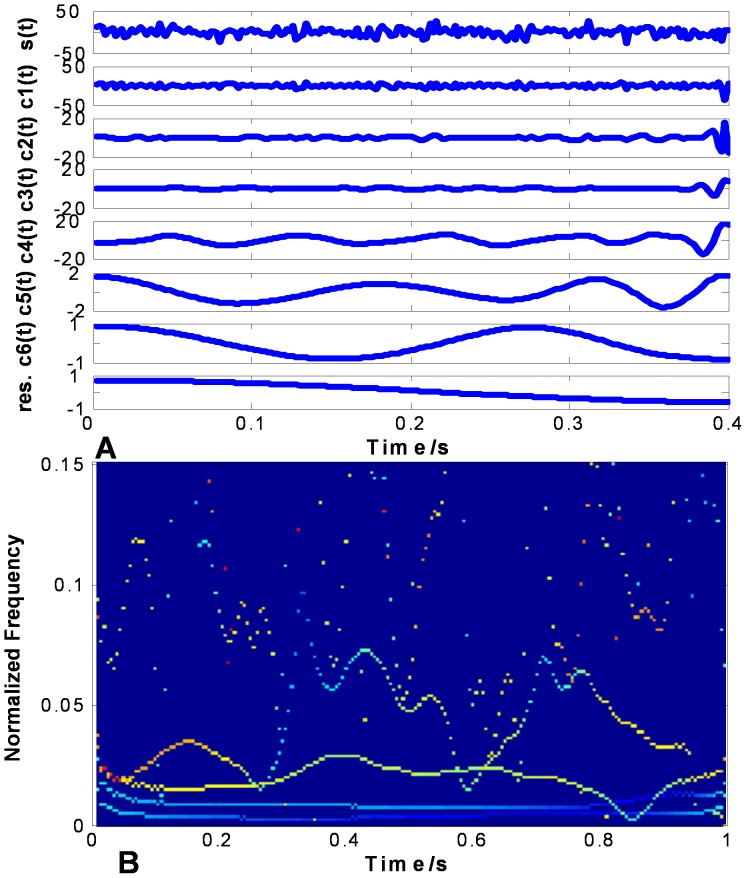
Random signal’s CSI EMD results. (a) shows random signal and its IMFs based on CSI, and (b) shows the corresponding Hilbert spectrum.

**Figure 11:**
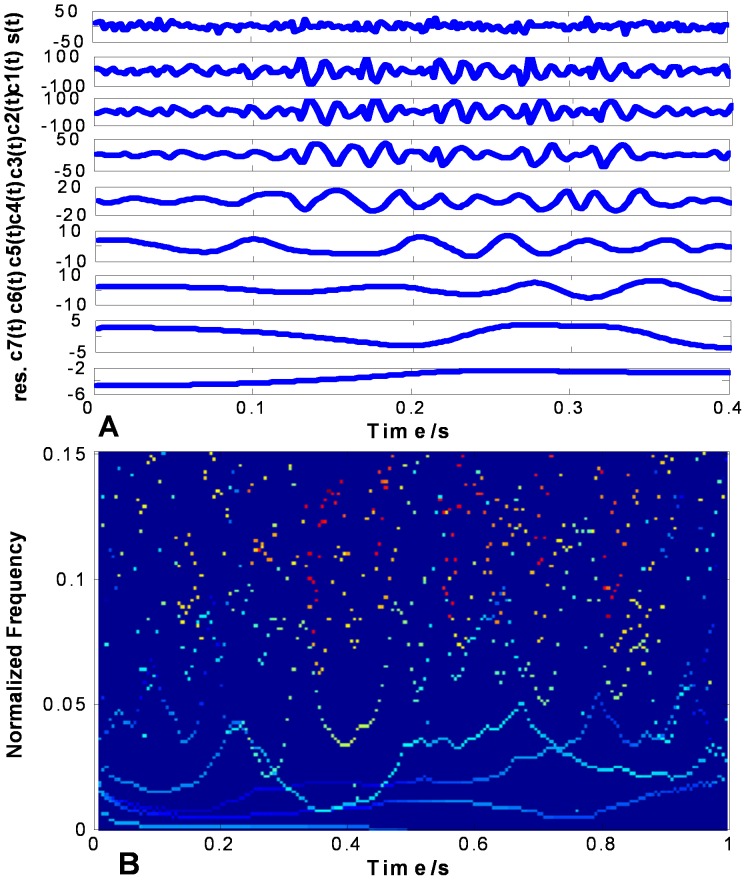
Random signal’s PPI EMD results. (a) shows random signal and its IMFs based on PPI, and (b) shows the corresponding Hilbert spectrum.

**Figure 12:**
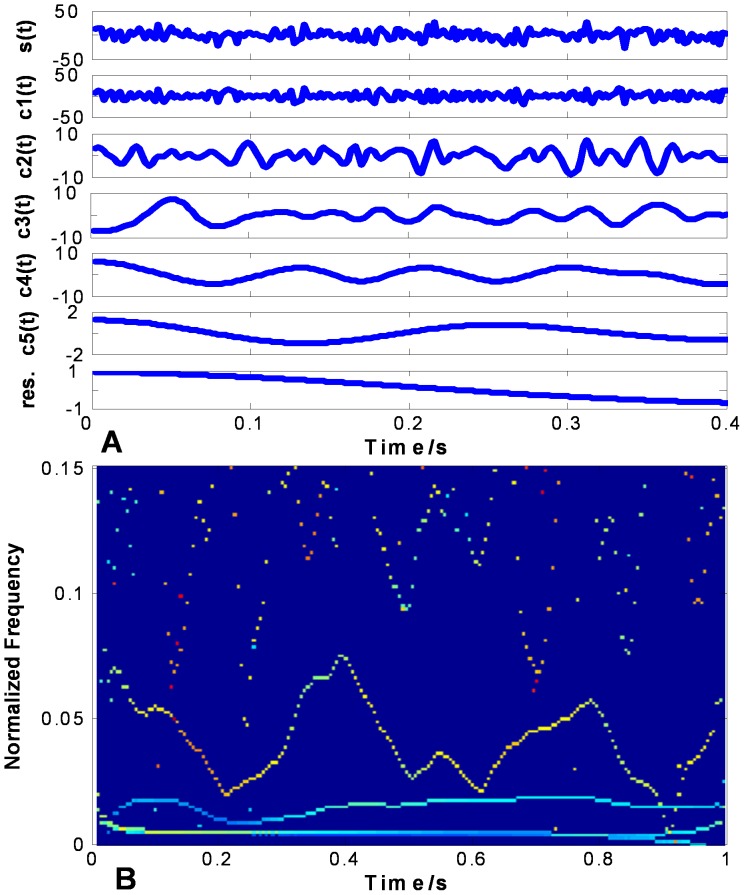
Random signal’s FC-PCHI EMD results. (a) shows random signal and its IMFs based on FC-PCHI, and (b) shows the corresponding Hilbert spectrum.

The Gaussian type random signal like 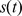 shown in the top of Figures 10 (a), 11 (a) and 12 (a) are used to evaluate the performance on non-stationary signal analysis. Figures 10 (a), 11 (a) and 12 (a) show the EMD results of 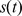 using CSI method, PPI method and FC-PCHI method, respectively. Figures 10 (b), 11 (b) and 12 (b) are the corresponding Hilbert spectrums with sampling frequency 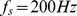. As we can see from Figure 10 (a), some high frequency components remain in the second IMF 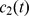 based on the CSI envelope. It suffers from one type of mode mixing problems that a single IMF is consisted of widely disparate scales [1]. It is shown as the undulating lines in the corresponding Hilbert spectrum. As the IMFs are based on the PPI envelope as shown in Figure 11 (a), the waveforms between 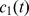 and 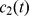, 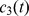 and 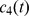 are similar. It suffers from another type of mode mixing problems that a signal is resided in different IMF components [1]. It is shown as the overlapping lines in the corresponding Hilbert spectrum. As shown in Figure 12 (a), the IMFs based on the proposed FC-PCHI envelope are quite different from each other, which illustrates the decomposition overcomes the mode mixing problem well.

100 random signals as mentioned above are decomposed using three methods, respectively. The average index of orthogonality (AIO), average index of energy conversation (AIEC) and standard deviations are compared in Table 2. For a statistical comparison, the t-statistic p-value is used. For IO, the t-statistic p-value is 4.31754E-15 between the FC-PCHI method and the CSI method and 8.09331E-22 between the FC-PCHI method and the PPI method, respectively. For IEC, the t-statistic p-value is 1.5091E-77 between the FC-PCHI method and the CSI method and 2.25373E-23 between the FC-PCHI method and the PPI method, respectively. All p-values are far less than 0.01, that is to say there are remarkable statistical differences between the FC-PCHI method and the CSI method and the FC-PCHI method and the PPI method for both IO and IEC test. Based on Table 2 and p-values, we can see that the proposed FC-PCHI method is the best among the three methods: not only for the AIO test but also for the AIEC test.

**Table 2:** The comparison of AIO and AIEC results for random signals.

	CSI method	PPI method	FC-PCHI method
AIO±SD	0.04456±0.03181	0.47267±0.27614	0.02222±0.01468
AIEC±SD	1.08353±0.06282	1.51106±0.31199	0.93509±0.03290

### 3.3 Real Signal: ECG data

The ECG data used in this study are from MIT-BIH Arrhythmia Database [18]. The database is a standard ECG database that includes typical ECG morphologies and typical kinds of noise. It contains 48 half-hour excerpts of two-channel ambulatory ECG recordings, obtained from 47 subjects studied by the BIH Arrhythmia Laboratory. Twenty-three recordings were chosen at random from a set of 4000 24-hour ambulatory ECG recordings collected from a mixed population of inpatients (about 60%) and outpatients (about 40%) at Boston’s Beth Israel Hospital; the remaining 25 recordings were selected from the same set to include less common but clinically significant arrhythmias that would not be well-represented in a small random sample. The recordings were digitized at 360 samples per second per channel with 11-bit resolution over a 10 mV range. Two or more cardiologists independently annotated each record; disagreements were resolved to obtain the computer-readable reference annotations for each beat included with the database.

**Figure 13:**
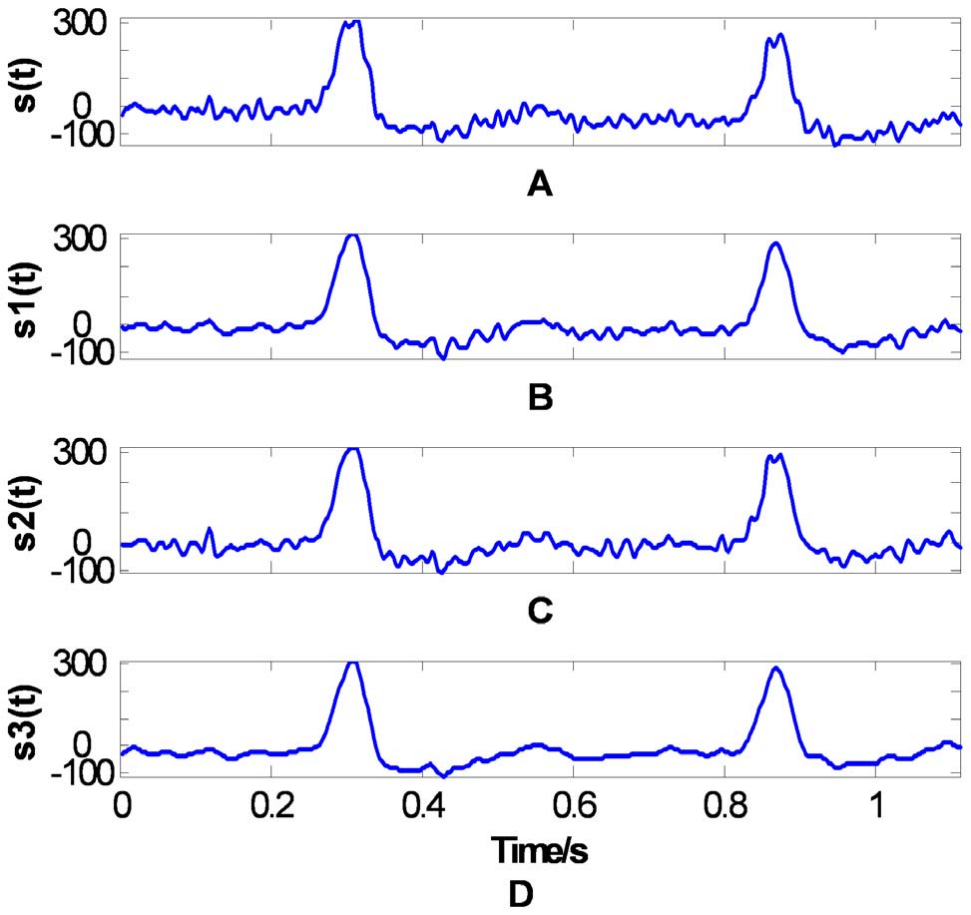
ECG signal and the reconstructed signals.

ECG signal is one type of weak biological signals. It is a typical non-stationary random signal. ECG signal is easily contaminated by high frequency noise such as electromyographic interference and low frequency noise such as baseline wander [19]. An original ECG signal from MIT-BIH Arrhythmia Database is shown in Figure 13 (a), which accompanies with severe high frequency noise and low frequency baseline tilt. As denoising is the first and important step in the ECG signal analysis system, it is necessary to have a simple and effective algorithm. In this paper, the EMD method is used to separate the high frequency noise and low frequency noise from the ECG signal.

**Figure 14:**
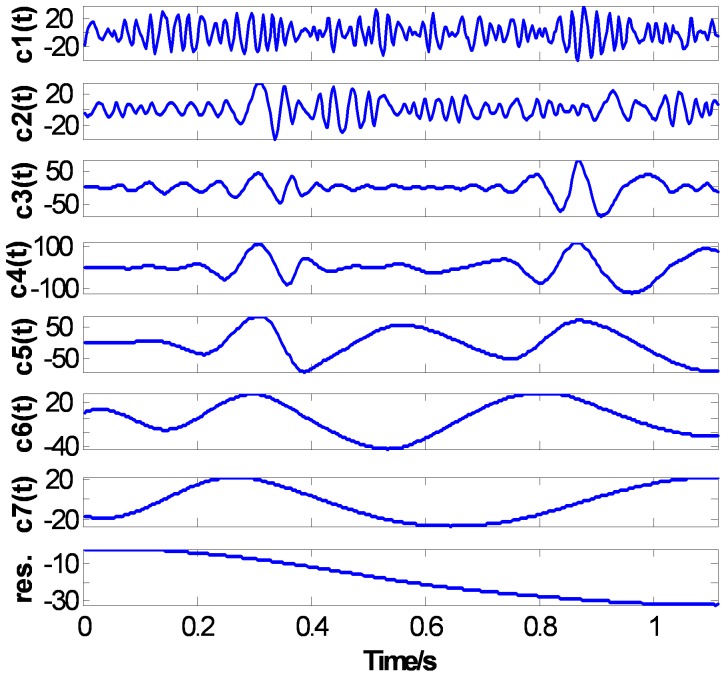
ECG’s CSI EMD results.

**Figure 15:**
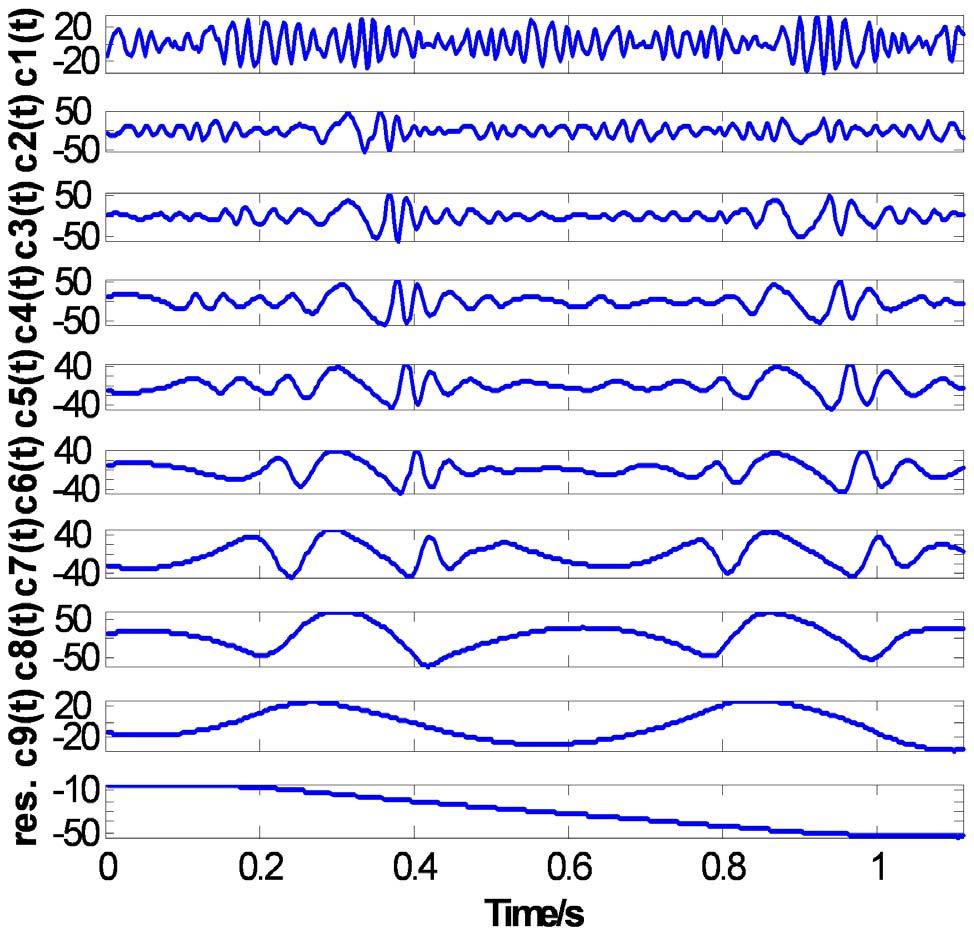
ECG’s PPI EMD results.

**Figure 16:**
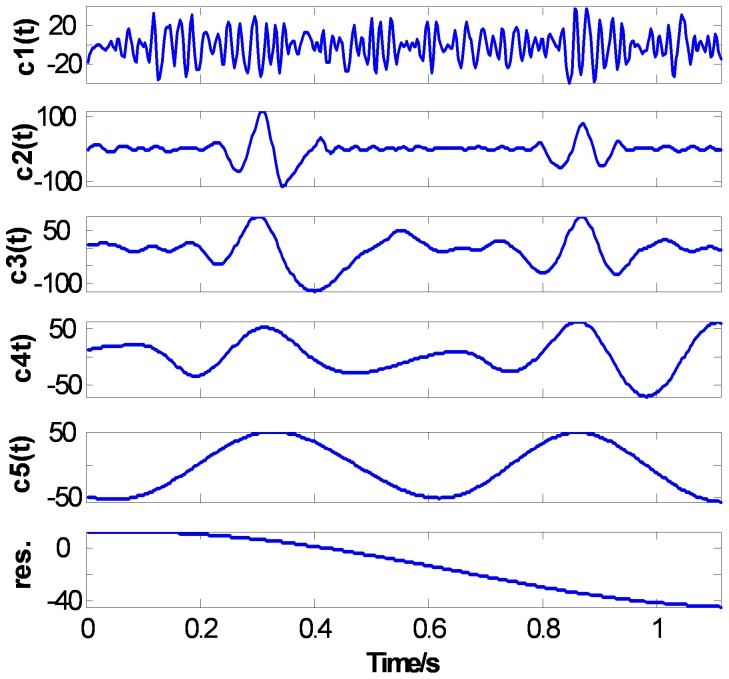
ECG’s FC-PCHI EMD results.

Figures 14, 15, and 16 show the EMD results of the ECG signal based on three methods: the CSI envelope, the PPI envelope and the proposed FC-PCHI envelope, respectively. As shown in the figures, the three first-order IMFs 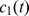 compose mainly high frequency noise, and the residuals are the ECG signals’ overall bias level. These noises should be removed from the original signal. As the IMFs with the CSI envelope method shown in Figure 14, the main component of the second-order IMF 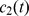 is still high frequency noise except some high frequency ingredient of the ECG signal at 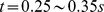. This means the noise and signal have not been separated well and the first type of mode mixing problem has occurred. The third-order IMF 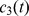 is also the admixture of great amount of signal and a small amount of noise. The IMFs 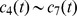 represent the comparatively pure ECG components.

The results of IMFs based on the PPI envelope method are shown in Figure 15. The phenomenon of admixture of signal and high frequency noise occur in both the second-order IMF 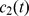 and the third-order IMF 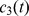. The IMFs 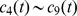 represent the comparatively pure ECG components. The waveforms between 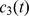 and 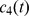, 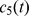 and 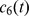 are similar. It suffers from the second type of mode mixing problems which a signal is resided in different IMF components.

The results of IMFs based on the proposed FC-PCHI envelope method are shown in Figure 16. The second-order IMF consists of mainly ECG component and accompanies some small amplitude high frequency noise which can be ignored. The IMFs 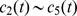 represent the components of the clean ECG signal.

By adding up the IMFs decomposed based on three methods except the first-order IMF and the residual respectively, the preliminary ECG denoising results with three methods are shown in Figure 13 (b), (c) and (d), respectively. As can see from Figure 13 (b) for the result of the CSI method, a small amount of high frequency noise still remains. The result of the PPI method is shown in Figure 13 (c), we can see that the remaining high frequency noise is obvious. While as shown in Figure 13 (d), the denoised signal by the proposed FC-PCHI method is the smoothest one.

200 random segments of ECG signals from all 48 records in the MIT-BIH Arrhythmia Database, which include typical ECG morphologies and typical kinds of noise, are decomposed using three methods, respectively. The comparison of AIO and AIEC results are listed in Table 3. In IO test, the t-statistic p-value is 5.94754E-06<0.01 between the FC-PCHI method and the CSI method, which means there is remarkable statistical difference between the two methods. The p-value is 0.0084<0.01 between the FC-PCHI method and the PPI method, which comes to the same conclusion. In IEC test, the t-statistic p-value is 0.0061<0.01 between the FC-PCHI method and the CSI method, which indicates the difference between the two methods is statistically significant. The p-value is 1.70268E-06<0.01 between the FC-PCHI method and the PPI method, which means there is remarkable statistical difference between the two methods. Based on Table 3 and the p-values, the proposed FC-PCHI method is the best among the three methods for both the AIO test and the AIEC test.

**Table 3:** The comparison of AIO and AIEC results for ECG signals.

	CSI method	PPI method	FC-PCHI method
AIO±SD	0.06328±0.05922	0.05430±0.04686	0.03953±0.04013
AIEC±SD	0.90014±0.02328	0.82030±0.16655	0.93041±0.00429

## Discussion and Conclusion

There are some interesting clues to the actual function of EMD that can be drawn based on both the CPSO method and the simulation examples. As we saw, CPSO method attempts to approach a set of the flattest upper and lower envelopes, and EMD performance appeared to be improved significantly. This may due to that EMD performs as a high-pass filter on the signal in each sifting iteration. This is possible since the local extrema, on which the sifting iteration is based, mainly carries information about the fast oscillating signal.

The interpolation method plays an important role in the sifting process. The PCHI is a local interpolation method in the sense that the corresponding piecewise polynomials depend only on the two nearest nodes. This renders the later interpolation method easier to handle mathematically than natural cubic spline.

In this paper, we proposed an alternative envelope fitting method for EMD, the FC-PCHI method. The analysis results of the proposed method on three different types of data: ascertain signal, random signals and real non-stationary signals showed that the proposed method has obtained much more accurate and reasonable results than those obtained by the classical CSI method and the PPI method.

In summary, our contributions are as follows:

1. The proposed FC-PCHI method effectively solves the overshoots caused by cubic spline interpolation (CSI) and the artificial bends caused by piecewise parabola interpolation (PPI).
2. In the IO test, the IO/AIO of the proposed method is 0.04054, 0.02222±0.01468 and 0.03953±0.04013 for the simulation signal, random signals and ECG signals respectively, which is lower than the IO/AIO of the IMFs based on either the CSI method or the PPI method, which means the IMFs are more orthogonal.
3. To get an idea of the energy conversation, the IEC or AIEC is adopted. The IEC/AIEC of the proposed method is 0.96193, 0.93509±0.03290 and 0.93041±0.00429 for the simulation signal, random signals and ECG signals respectively. It is closer to 1 than the IEC/AIEC of the IMFs based on both the CSI method and the PPI method, which indicates the total energy deviation amongst the components is smaller.
4. The comparisons of the Hilbert spectrums show that the proposed method overcomes the mode mixing problems very well, and make the instantaneous frequency more physically meaningful.

However, if there are many extreme points in a data, the CPSO may take a long time. This will affect the pace of the EMD. That is to say, the proposed FC-PCHI method is not suitable for long and very noisy data. The research toward the reduction of the time cost for the proposed FC-PCHI method is still in the investigation.

In addition, as the interpolation procedure may lose original information or create additional information which has nothing to do with the original data, the inherent problems during the SP such as end issue, overshoot and mode mixing may be caused by the interpolation issues. The interpolation may make the SP and corresponding IMFs depending on the interpolants. Therefore, free-interpolation models for SP are also our future research interests.
